# A comprehensive review of massive transfusion and major hemorrhage protocols: origins, core principles and practical implementation

**DOI:** 10.1016/j.bjane.2024.844583

**Published:** 2024-12-25

**Authors:** David Silveira Marinho, Denise Menezes Brunetta, Luciana Maria de Barros Carlos, Luany Elvira Mesquita Carvalho, Jessica Silva Miranda

**Affiliations:** a Serviço de Anestesiologia, Instituto Doutor José Frota; Unidade de Transplante Hepático, Serviço de Anestesiologia, Hospital Geral de Fortaleza, Fortaleza, CE, Brazil; b Centro de Hematologia e Hemoterapia do Ceará (HEMOCE); Empresa Brasileira de Serviços Hospitalares (EBSERH); Departamento de Cirurgia, Faculdade de Medicina, Universidade Federal do Ceará, Fortaleza, CE, Brazil; c Centro de Hematologia e Hemoterapia do Ceará (HEMOCE); Núcleo Transfusional, Instituto Doutor José Frota, Fortaleza, CE, Brazil; d Division of Cardiothoracic Anesthesia, Department of Anesthesiology, Perioperative and Pain Medicine, Mount Sinai Hospital; Assistant Professor, Mount Sinai School of Medicine, New York, NY, USA

**Keywords:** Blood component transfusion, Blood coagulation disorders, Exsanguination, Hemostasis, Hemorrhage, Shock, hemorrhagic

## Abstract

Until the beginning of the century, bleeding management was similar in elective surgeries or exsanguination scenarios: clotting tests were used to guide blood product orders and, while awaiting these results, an aggressive resuscitation with crystalloids was recommended. The high mortality rate in severe hemorrhages managed with this strategy endorsed the need for a special resuscitation plan. As a result, modifications were recommended to develop a new clinical approach to these patients, called “Damage Control Resuscitation”. This strategy includes four principles: damage control surgery, minimization of crystalloids, permissive hypotension and hemostatic resuscitation. The latter involves the use of antifibrinolytics, correction of preconditions of hemostasis (calcium, pH and temperature) and the early and rapid restoration of intravascular volume with blood products. To enable timely availability and transfusion of blood products, specific actions in different hospital areas need to be synchronized, which are usually organized through Massive Transfusion Protocols or, as they have recently been rebranded, Major Hemorrhage Protocols (MHPs). Although these bundles of actions represent a paradigm change, essential aspects such as their historical evolution, theoretical foundations, terminology and operational elements have yet to be well explored. Considering the wide application range of these tools (emergency departments, interventional radiology, operating rooms and military fields), it is essential to integrate all professionals involved with severe hemorrhage scenarios in the implementation of the aforementioned protocols, from conception to execution and management. This review paper addresses MHP aspects relevant to anesthesiologists, transfusion services and other areas involved with the care of patients with severe bleeding.

## Introduction

Severe bleeding is the leading cause of preventable early death by trauma^1^ and maternal mortality.^2^ Fewer than 10% of trauma victims need transfusions, and less than half of these victims receive massive amounts of blood.^3^ However, this small group consumes more than 70% of blood products in trauma centers and accounts for most preventable hemorrhagic deaths.^4^^,^^5^ The unique challenges in caring for these patients have led to a management strategy with Major Hemorrhage Protocols (MHPs) as a key pillar.

Despite its relevance, Brazilian hematology and anesthesiology journals have few publications on this topic, with only a few reports in which such protocols were used.^6^^,^^7^ Thus, essential aspects, such as their historical evolution, terminology and operational elements, still need to be thoroughly explored. This review aims to help fill these gaps by using adult trauma-related bleeding as a prototypical scenario and the blinded use of transfusion packs as the standard source of blood products.

## Glossary of terms

Blood products: Whole Blood (WB) and Blood Components (BC): Red Blood Cells (RBC), Platelet Concentrate (PC), Fresh Frozen Plasma (FFP) and cryoprecipitate; obtained by donation and used for transfusion.

Blood elements: Red blood cells, platelets and clotting factors as blood constituents.

Massive transfusion protocols: Structured multidisciplinary actions designed to manage exsanguinating situations effectively. This has been renamed “Major hemorrhage protocols”.

Transfusion packs: Sets of BCs jointly and cyclically released according to the local MHP.

Damage Control Surgery (DCS): A strategy encompassing emergency surgical procedures that prioritize patient survival over immediate definitive treatment.

Damage Control Resuscitation (DCR): Set of actions aimed at addressing exsanguinating scenarios that combines the principles of DCS, Permissive Hypotension (PH), minimization of crystalloids and hemostatic resuscitation.

Hemostatic Resuscitation (HR): Set of actions of DCR aimed at preserving the integrity of hemostasis.

## Massive transfusion, major bleeding management and MHPs

In the 1950s, reports on transfusion demands that align with today's concepts of “Massive Transfusion” (MT) began to emerge;^8^ however, it was only in the latter half of the 20^th^ century that the DCS approach was introduced, which transformed strategies for managing trauma and critical bleeding.^9^ The term “damage control”, originating from the U.S. Navy, describes emergency actions to prevent shipwreck without aiming for permanent repairs, allowing mission completion. In hemorrhagic risk scenarios, it was adapted to refer to emergency surgeries focused on survival rather than immediate definitive injury treatment.^9^^,^^10^ DCS reduces major bleeding mortality, but resuscitation remains inadequate. Efforts have focused on hemodynamic stability, acidosis reversal, and hypothermia prevention, whereas coagulopathy has been overlooked and is viewed as an inevitable outcome of resuscitation, hypothermia, and delays in blood administration.^11^

Until the second world war, patients with major bleeding received the only blood product available: WB.^12^ WB fractionation techniques were only developed in the 1950s, with the aim of improving the effectiveness of logistics, cost, and supply optimization.^13^ With laboratory results and predefined trigger values, it would be possible to transfuse only the BC in deficit. In this way, between the late 1950s and the mid-1980s, WB and BC coexisted throughout Transfusion Services (TSs), and the support strategy varied according to the blood product available.^13^

Since it seems intuitive and harmless, BC use has become the preferred approach despite the lack of proper scientific analysis.^14^ As a result, in the last two decades of the 20^th^ century, WB was unavailable in most TSs, and the indications for its use were restricted, controversial^15^ or nonexistent, as it was in Brazil.^16^

**Figure 1:**
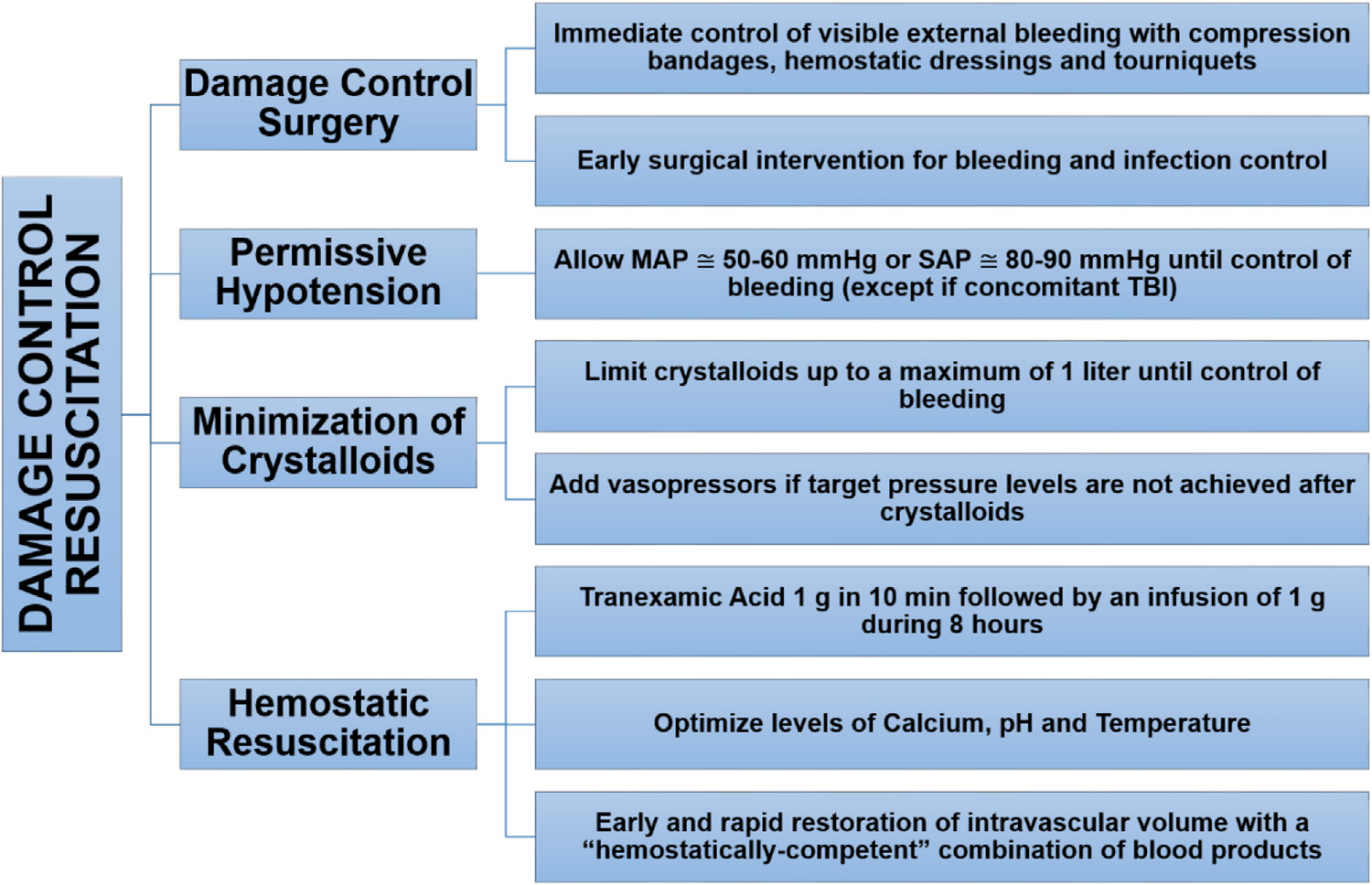
Damage Control Resuscitation, its pillars and proposed actions. MAP, Mean Arterial Pressure; SAP, Systolic Arterial Pressure; TBI, Traumatic Brain Injury.

In the 2000s, military data^17^^,^^18^ demonstrated that failures and delays in the management of coagulopathic bleeding had become the leading cause of trauma-preventable deaths.^11^ Additionally, complications from fluid overload (abdominal compartment syndrome and dilutional coagulopathy) have become frequent, and the mortality of patients requiring MT has exceeded 80%.^19^ These observations combined with data on the resuscitation approach in military scenarios changed the management of severe bleeding.^4^^,^^11^^,^^20^ When exsanguination risk arose, DCS was combined with three principles ‒ “Permissive Hypotension”, “Minimization of Crystalloids”, and “Hemostatic Resuscitation” ‒ forming the DCR strategy, which became the new paradigm for managing major hemorrhage (Fig. 1).^4^^,^^9^^,^^11^

In 2005, an international conference reviewed early MT in trauma and discussed blood product options for resuscitation.^21^ Despite its limited availability, WB was deemed the best choice for maintaining hemostatic and oxygen transport functions, even if it completely replaced the patient's blood volume.^15^ The obvious solution to the insufficient availability of WB was the combination of PCs, FFP and RBC to “reconstitute” WB, maintaining a relatively fixed proportion between the components and organizing the resuscitation efforts in blocks repeated cyclically until stabilization or death.^22^, ^23^, ^24^, ^25^, ^26^, ^27^ Therefore, in exsanguinating, it was proposed that waiting for test results to guide transfusion was unnecessary since the need for all blood elements would be irrefutable.^28^ A fixed “optimal proportion” of BC would provide an adequate composition for both hemostasis and oxygen transport. Empirical use of FFP and PC supplementation bypassed delays in laboratory results. Additionally, the cyclic predictable nature of transfusion demands could result in faster preparation and release of blood products.

By the end of the 2000s, despite promising results with this new strategy, trauma centers in German-speaking countries progressively developed another solution to the problems associated with traditional resuscitation. They proposed a replacement strategy incorporating viscoelastic test results (such as thromboelastometry) and clotting factor concentrates, allowing for rapid interventions based on the specific needs of each patient.^29^, ^30^, ^31^ However, the best approach for managing bleeding and coagulopathy in severe trauma remains controversial.^32^

## Damage control resuscitation

PH suggests limiting resuscitation to a *systolic* Blood Pressure (BP) of 80–90 mmHg to reduce mechanical stress, support the stability of newly formed clots, and limit crystalloid infusion.^33^ However, in severe traumatic brain injury, cerebral perfusion should be prioritized, maintaining a *mean* BP ≥ 80 mmHg.^34^

Minimization of crystalloids is recommended to limit these fluids to one liter until bleeding is controlled^35^ to minimize hemodilution and other adverse effects. If such a volume fails to achieve the target BP, transient combination with vasopressors is acceptable.^34^ A recent meta-analysis concluded that, compared with patients receiving conventional fluid resuscitation, patients receiving hypotensive fluid resuscitation experienced lower mortality rates (12.5% vs. 21.4%; RR = 0.58; 95% CI 0.51–0.66; p < 0.001) and fewer adverse events (10.8% vs. 13.4%; RR = 0.70; 95% CI 0.59–0.83; p < 0.001).^36^ Additionally, while pH is crucial *until* bleeding is controlled, it may be associated with myocardial injury.^37^ Thus, it is vital to limit the duration of hypotension and ensure that the mean BP remains above 60–65 mmHg once bleeding is halted.

HR is intended to resuscitate the patient while preserving the integrity of hemostasis (or even reversing deficits that may already be present).^33^ Among HR practices, the normalization of coagulation preconditions (temperature, pH and calcium) always plays a crucial role.^4^^,^^11^ In contrast, there is initially limited evidence supporting the use of Tranexamic Acid (TXA) in trauma.^38^ However, with improved understanding of trauma coagulopathy and positive trial results, early TXA administration has become central to HR strategies.^39^^,^^40^

TXA should be promptly administered, but only if trauma occurs within three hours. A meta-analysis of randomized trials involving trauma patients revealed that the effect of TXA on survival was comparable between non-severely injured patients (OR = 1.25, 95% CI 1.03–1.50) and those with severe injuries (OR = 1.22, 95% CI 1.09–1.37). In both groups, treatment within the first hour after injury was the most effective.^41^ Despite the theoretical risk of complications associated with TXA, it is considered safe for bleeding patients. A meta-analysis revealed no evidence of an increased risk of thrombotic events (RR = 1.00 [95% CI 0.93–1.08]), seizures (RR = 1.18 [95% CI 0.91–1.53]), venous thromboembolism (RR = 1.04 [95% CI 0.92–1.17]), acute coronary syndrome (RR = 0.88 [95% CI 0.78–1.00]) or stroke (RR = 1.12 [95% CI 0.98–1.27]) associated with its use.^42^ Epsilon-Aminocaproic Acid (EACA) has similar mechanisms of action and can be used if TXA is unavailable at a bolus dose of 150 mg.kg^−1^, followed by 15 mg.kg^−1^.h^−1^ infusion until risk reduction.^34^ However, in addition to the higher cost of EA, there is insufficient evidence on the benefit of EACA for traumatic bleeding.^43^

Hypovolemia itself (and not anemia or coagulopathy) is the primary cause of circulatory collapse and death in severe hemorrhages.^44^ For this reason, despite the importance of administering TXA and correcting hemostatic preconditions, the main goal of HR is the early restoration of intravascular volume with a product that does not induce a deleterious dilutional effect on blood composition.^45^

Considering an “adequate” product as one with hematocrit > 21%, platelet count > 50,000 μL, fibrinogen > 100 mg.dL^−1^, and other coagulation factors at concentrations above 50% of their original levels (approximate INR ≅ 1.5),^46^ only a few ratios, specifically between 1:1:1 and 1:1:2, proved to meet these standards. However, using proportions with marginal blood elements risks dilution from nonblood fluids, which, even in small amounts, can cause deficiencies in one or more components (see Table 1 and the Supplementary Material).^11^

**Table 1:** Mutual dilutional effect of blood components in different combinations. As a patient severely bleeds and continuously receives one of the associations above, the levels of blood elements progressively assume the hematologic profile of the final reconstituted product. This way, theoretically, a patient who had its whole blood volume substituted by one of the combinations above would acquire the concentrations shown above. By assuming the final product adequation criteria specified in the article, values highlighted in bold show insufficient levels, whereas those in italic demonstrate unpurposely high levels. Definitions, characteristics of blood components and formulas used to generate this table are available in supplementary material, including the possibility of simulating results with other characteristics or combinations.

Combinations					Hematologic profile of the final reconstituted product			
FFP	PC	RBC	Cryo	Fluids_(mL)_	FactorsConc._(%)_	Ht_(%)_	Plt_(/µL)_	Fib_(mg.dL_^−1^_)_
1	1	1	0	0	60	28	88.000	164
1	1	2	0	0	50	37	57.895	137
1	1	3	0	0	44	42	43.137	121
1	2	1	0	0	62	26	162.963	169
2	1	1	0	0	67	20	62.857	183
2	2	1	0	0	68	19	118.919	185
1	2	2	0	0	53	36	110.000	143
1	1	1	1	0	58	28	85.938	213
1	1	2	1	0	49	37	56.995	175
1	1	1	0	500	28	16	48.889	77

FFP, Units of Fresh Frozen Plasma; PC, Units of Platelet Concentrates (single); RBC, Units of Red Blood Cells Concentrates; Cryo, Units of Cryoprecipitate; FactorsConc.: Final percent of clotting factors in comparison with the concentration of the original donated plasma; Ht, Hematocrit; Plt, Platelet count; Fib, Fibrinogen level.

Beyond this mathematical analysis, studies of small cohorts suggested that 1:1:1 combinations may yield better outcomes, although the results were not statistically significant.^47^, ^48^, ^49^ Computer models simulating severe bleeding and hemodilution have consistently recommended ratios between 1:1:1 and 1:1:2.^50^, ^51^, ^52^ Studies in both military^53^ and civilian^54^ settings have demonstrated benefits of these proportions.

Over the past decade, key aspects of DCRs have led to recommendations for hospitals to implement MHPs, including clear, practical recommendations and training for all the involved professionals.^55^

## MHPs: Terminology

### Massive transfusion vs. major bleeding

Wilson et al. were the first to objectively define MT as ten or more WB units (approximately one blood volume) within 24-hours,^56^ a time frame likely chosen for data collection convenience.^57^ Due to WB scarcity in the 1980s, adaptations with questionable equivalence emerged, such as requiring ten or more RBCs in 24 hours (grossly equivalent to the erythrocyte volume of the original definition)^47^^,^^58^ or total transfusion exceeding one blood volume in 24-hours.^59^ Although definitions adopting shorter periods have been proposed,^60^ their wide time interval severely limits their applicability.

With respect to DCR, identifying patients *at risk* of MT, rather than waiting for MT criteria, was crucial. Therefore, the definition of MT evolved to align with major bleeding. Accordingly, *nonmassive* quantities of *rapidly* transfused products (≥ 3‒4 RBCs in one hour^61^^,^^62^ or, more recently, ≥ 4 RBCs in two hours),^63^ rapid blood loss (≥ 150 mL.min^−1^ or ≥ 50% of blood volume loss in three hours)^64^ and clinical scores were incorporated to identify severe bleeding and patients *at risk* of MT. Some of these criteria are now adopted as DCR triggers (see below).

“Massive Transfusion Protocol” vs. “Major Hemorrhage Protocol”

Many Brazilian institutions recently adopted the term MTP, a straightforward translation of its English counterpart. However, this translation can lead to misconceptions, such as, once the protocol is activated, the only action required would be to transfuse blood products (and, necessarily, in massive amounts). Notably, MTs are not a target themselves (the goal of transfusions is to restore intravascular volume with blood products in appropriate proportions and quantities, regardless of whether they are massive). For this reason, the expression “Major Hemorrhage Protocol”^65^ may provide more intelligibility by informing that transfusion support ‒ not necessarily massive ‒ is just part of a broad set of actions.

### MTPs: activation criteria, activation processes and teamwork

Until the early 2000s, MHPs were activated based on vague criteria (“severe, difficult-to-control bleeding” or “seriously injured patients”) or as a routine response after a number/rate of transfused RBCs.^20^ With respect to DCR, clinical, laboratory and ultrasound criteria are integrated into scores to predict outcomes such as MT demand, coagulopathy development, and hemorrhagic death.^66^^,^^67^

Publications about these scores and their predictive performance, advantages and disadvantages are readily available.^66^^,^^67^ Overall, as more variables are added, the accuracy of the score improves; however, this also increases the time needed to collect data, potentially delaying the first transfusion.^68^ Two widely used scores are the “*Shock Index*”, which includes only vital signs,^69^ and the “*ABC (Assessment of Blood Consumption)*”, which also incorporates ultrasound evaluation and trauma mechanisms (Table 2).^70^ Although tempting, unrestricted use of these scores may be inadequate, as they were validated primarily for trauma. They are meant to supplement, not replace, clinical judgment, which alone has poor predictive performance.^71^ In trauma, blood loss estimation can be inaccurate because of limited scene information. Nevertheless, the Advanced Trauma Life Support® Manual has updated its recommendations to include “class IV hemorrhage” (blood loss greater than 40% of blood volume) as a trigger for MHP activation.^35^

**Table 2:** Examples of triggers and usual cutoffs for DCR and MTP's.

Trigger	Definition	Usual cutoffs
Shock Index		≥ 0.8‒1.0
Assessment of Blood Consumption (ABC)	1-point for each: SAP ≤ 90 mmHg; HR ≥ 120 bpm	≥ 2-point
	Positive FAST (free fluid in any of the following regions: pericardium, perihepatic area, perisplenic area or pelvis)	
	Penetrating trunk injury	
Critical Administration Threshold (CAT)	Number of RBCs in any 60-minute-interval	≥ 3-units
Resuscitation Intensity (RI)	1-point for every intervention below in the first 30 minutes after admission: 1L of crystalloids; 0.5 L of colloids; 1 unit of RBC; 1-unit of FFP; 6-single (random) PC's	≥ 4-points

HR, Heart Rate; SAP, Systolic Arterial Pressure; bpm, Beats per minute; FAST, Focused Assessment with Sonography for Trauma; RBC, Red Blood Cell Concentrate; FFP, Fresh Frozen Plasma; PC, Platelet Concentrates.

MHPs can also be activated by the degree of resuscitation (Table 2). While some measures have been validated only for the initial minutes of care, they can coexist with predictive scores and act as triggers throughout resuscitation for patients who have not yet reached critical MHP activation thresholds.^62^^,^^72^^,^^73^

**Figure 2:**
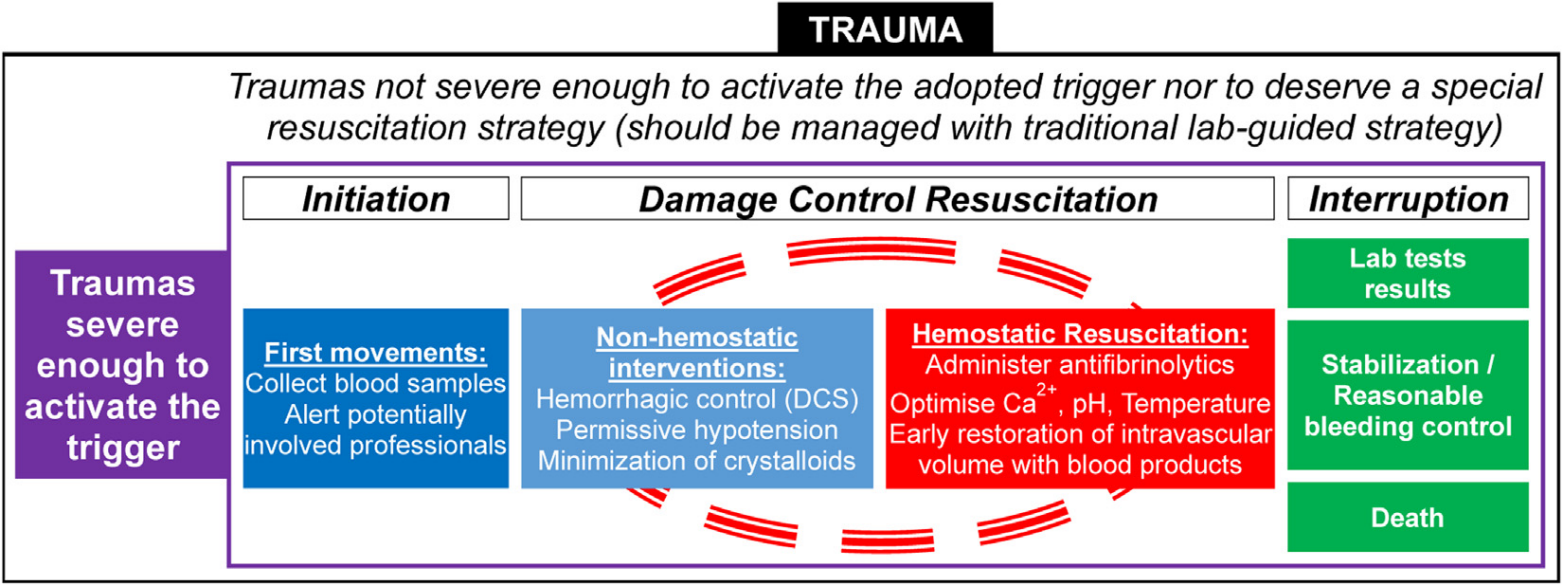
General organization of actions during Damage Control Resuscitation. Among trauma-related hemorrhages, only a few are severe enough to activate the commonly used triggers for DCR. Most bleedings should be managed using traditional lab-guided strategy. Severe hemorrhages activate a series of actions involving first movements followed by a cycle of non-hemostatic interventions in parallel to hemostatic resuscitation, which are maintained until any of the interruption criteria arrives. DCS, Damage Control Surgery; Ca^2+^, Ionised Calcium.

**Figure 3:**
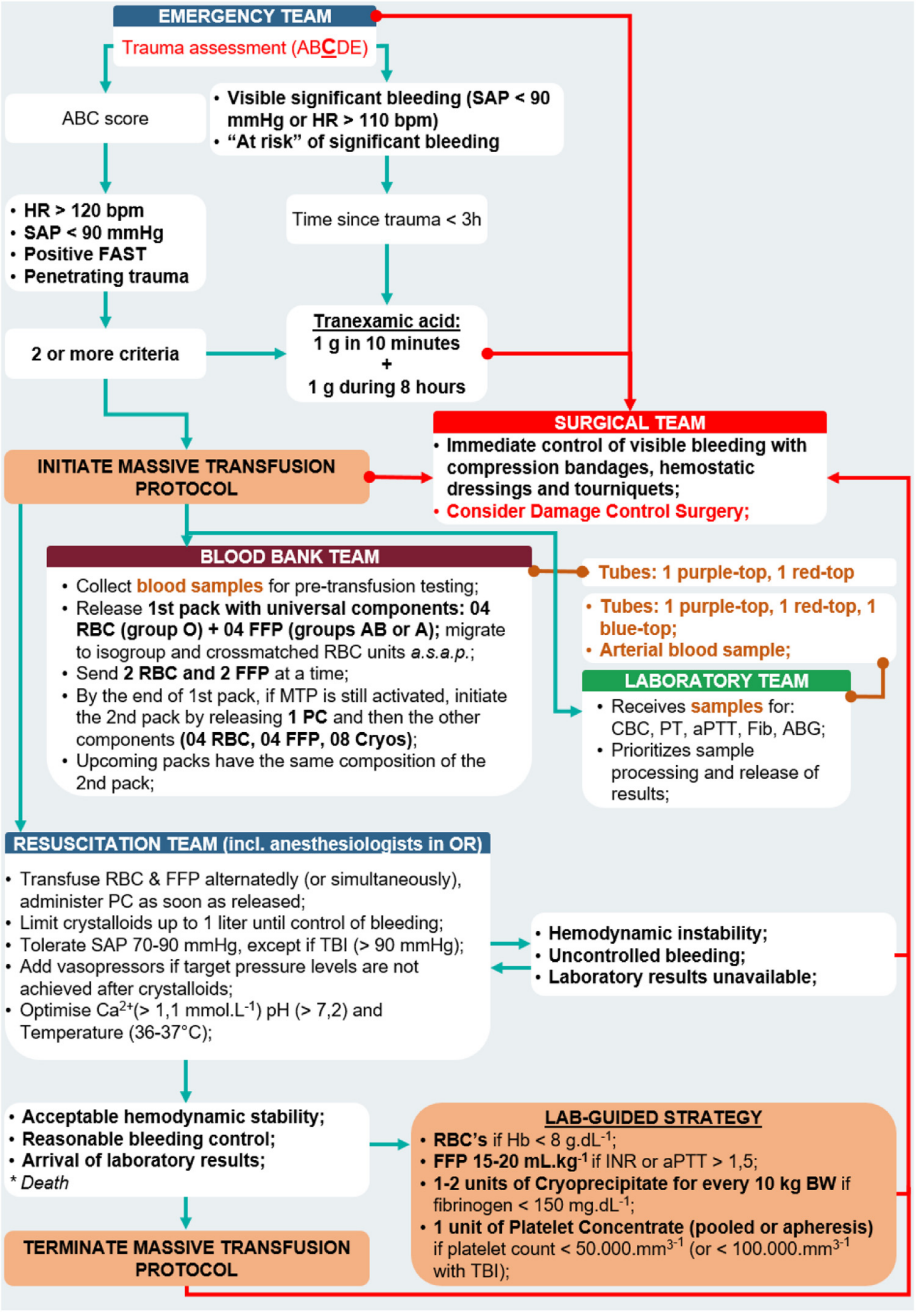
Example of MTP in a Level-1 Trauma Center (Instituto Dr. José Frota, Fortaleza-CE, Brazil). HR, Heart Rate; SAP, Systolic Arterial Pressure; bpm, beats per minute; FAST, Focused Assessment with Sonography for Trauma; RBC, Red Blood Cell Concentrates; FFP, Fresh Frozen Plasma; PC, Platelet Concentrate; MTP, Massive Transfusion Protocol; Cryo, Cryoprecipitate; CBC, Complete Blood Count; PT, Prothrombin Time; aPTT, Activated Partial Thromboplastin Time; TBI, Traumatic Brain Injury; Ca^2+^, Ionized Calcium.

Once activated, several simultaneous actions are triggered.^65^ All hospital departments involved in resuscitation, particularly the laboratory, TS, and operating theater (or interventional radiology), should be promptly notified. The samples should be collected, completely identified and sent to the lab and TS without delay. Additionally, the principles of DCR should be implemented (Figure 2, Figure 3).

A designated team leader ‒ typically the most senior doctor on site ‒ should be quickly appointed to direct and coordinate management. This professional must have strong teamwork skills, leadership abilities, situational awareness and the capacity to make decisions under pressure.^74^

HR typically begins with prompt TXA infusion at an intravenous dose of 1g in 10 minutes, followed by 1g over eight hours.^75^ Temperature, pH, and calcium are corrected along with the restoration of intravascular volume through transfusion. Preferably, RBCs and FFPs should be transfused alternately^76^ to provide a continuously balanced supply. PCs are usually included from the second pack forward and should be the first transfused product of the pack in which they are included.^76^ Importantly, the 1:1:1 or 1:1:2 proportions refer to single, individual, or random PCs (i.e., one platelet unit obtained by centrifuging the WB bag of a single donor). Apart from the pediatric setting, the common practice in Brazil is to release one pool of PCs obtained from 4–6 donors or one apheresis PC (one unit from one donor but equivalent to 5–6 single PCs).^16^ However, as the platelet count is usually rapidly available, blind PC transfusions may be unnecessary and should be guided by laboratory results whenever possible. Three events should modulate (or terminate) the blind transfusion support: death, hemodynamic stabilization and the release of lab results.^65^^,^^77^

We must emphasize that the goal of combining BCs during MHP is not to achieve complete normalization of hemodynamics or to stop bleeding.^74^ Its primary aim is to restore intravascular volume with a blood product that does not lead to a deleterious dilutional effect on hemostasis or oxygen transport, ensuring minimum BP to prevent hemorrhagic death until bleeding is controlled. Accordingly, anesthesiologists and surgeons should be aligned about not delaying procedures while waiting for hemodynamic improvement or complete stabilization.^34^ Therefore, it is vital to involve appropriate expertise to address the bleeding source or proceed to early referral, if necessary. In more challenging cases, early action may be necessary to pack the visceral cavities and cross-clamp and tie off major vessels. Radiological embolization and/or stenting can also have roles in certain clinical situations. An Intensive Care Unit (ICU) bed may be needed, and early communication is advisable to ensure availability.^74^

Protocol actions must be maintained by bedside and support teams, including during transport and procedures, until MHP deactivation. Every 30–60 minutes, hemoglobin, blood gas analysis, lactate, calcium, platelet count, PT, aPTT, and fibrinogen (by the Clauss method) assessments should be considered a minimum standard because each patient's needs can fluctuate both individually and over time.^78^ Furthermore, the use of point-of-care devices to assess hemostasis is expanding, as they provide rapid results, add diagnostic capacity for problems not detected by conventional methods (such as fibrinolysis) and assess the interaction between the elements of hemostasis.^79^

For the successful implementation of MHPs, it is crucial to manage major hemorrhage with the same principles applied to any medical emergency.^78^ Key factors include effective communication and coordination to optimize the delivery of safe and effective responses and resource utilization. These are essential to prevent poor clinical outcomes, suboptimal or inappropriate transfusions and component waste.^74^ Furthermore, transfusion specialists should be involved in patient-specific consultations and in establishing hospital policies for the prevention and management of major hemorrhage.^79^

MHP is deactivated when satisfactory control of bleeding is achieved. Such events must be communicated to the involved departments, and the unused units should be returned. Subsequently, residual mild defects in hemostasis may be managed according to laboratory results.^55^

Following MHP deactivation, teams should hold debriefs to address learning points from the event, including providing emotional support for staff as needed.^78^ Even in advanced trauma systems, preventable deaths can occur.^80^ Audits can reveal specific areas of improvement and error patterns, providing opportunities for targeted policy interventions.^80^

### MHPs: TS and laboratory roles

**Figure 4:**
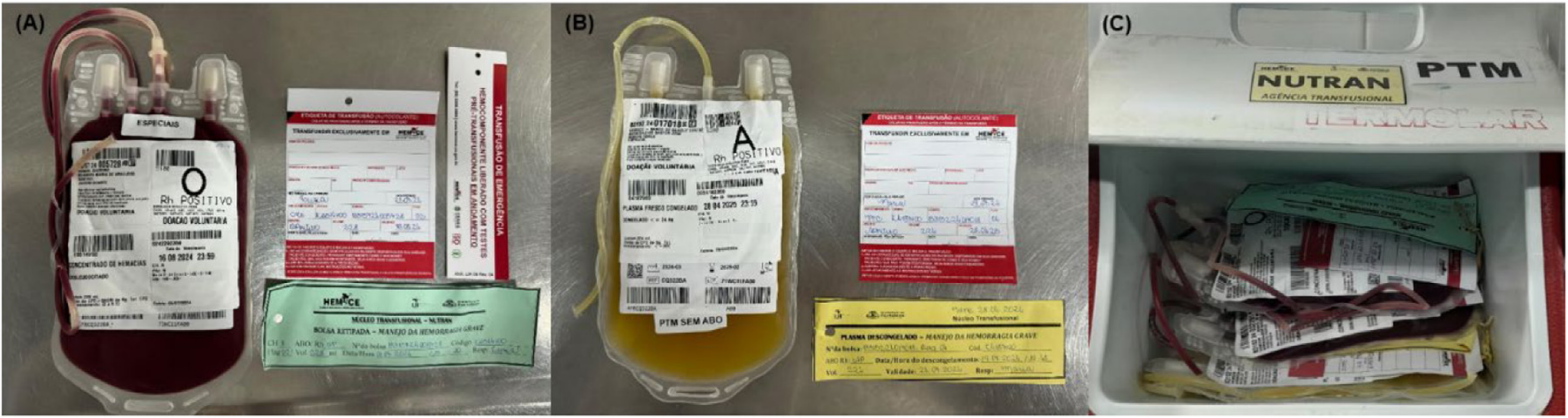
Transfusion packs in a MTP in a Level-1 Trauma Center (Instituto Dr. José Frota, Fortaleza-CE, Brazil). (A and B) Release of blood components may be expedited by the conservation of group-O RBC previously retyped with a negative direct antiglobulin test and marked with a transfusion emergency label partially filled in; (C) Example of a first package of MTP containing RBC and FFP units in a cooler box, according to local protocol (thawed plasma is available 24h a day).

Brazilian law requires protocols for the rapid release of blood products in urgent situations, even before full pretransfusion testing, known as “emergency transfusions”.^81^ In addition to implementing a protocol discussed and approved by the local transfusion committee, certain measures can help expedite the release of initial BC. These include storing thawed plasma around the clock and maintaining a supply of group-O RBCs that have been retyped, confirmed to have a negative direct antiglobulin test, and prelabeled for emergency transfusion with partially completed information (Fig. 4).

Once notified, the TS immediately releases previously prepared group-O RBCs or WBs, prioritizing RhD-negative units for childbearing-aged women and RhD-positive units for other patients. In cases of limited O RhD-negative supply, O RhD-positive blood may be administered to all patients, as the minimal risks associated with RhD-positive blood are outweighed by its critical, lifesaving benefits.^82^ Pretransfusion testing should also commence. A critical factor in this workflow is the prompt arrival of a clearly labeled sample at the TS, which must include the patient's full name (without abbreviations), date of birth or hospital ID, name of the professional responsible for the collection, and collection date.^81^ For unknown patients, the sample should be labeled using the institution's established ID protocol as a substitute for the patient's name. This ensures that pretransfusion testing, such as blood typing and crossmatching, can proceed without delay, enabling efficient management of emergency transfusion cases and enhancing patient safety.

The release of FFPs and PCs in the first package may vary (Table 3).^83^, ^84^, ^85^, ^86^ Institutions with a high transfusion demand can maintain the continuous availability of thawed plasma units (kept at 2–6°C), which can be sent immediately upon MHP activation, facilitating adherence to ratios closer to 1:1. If there is no activation, those units can be used in usual hospital plasma requests, with an expiring shelf life of 24-hours after thawing.^16^ However, there is evidence indicating that clotting factors may remain stable in thawed plasma stored for up to 5-days at 2–6°C.^78^^,^^87^ This extended stability can reduce the likelihood of wastage, making it a practical option for hospitals with moderate emergency transfusion needs. When such units are used, new bags are thawed for possible future activation. Thus, in larger hospitals, it is common for the first cooler to contain FFPs and RBC units at ratios between 1:1 and 1:2. Hospitals with lower transfusion demands often start the protocol by sending only group-O RBCs and simultaneously initiating the thawing of FFPs to be sent later. A possible disadvantage of such a format is initial “unbalanced” resuscitation due to the absence of FFP. However, subsequent coolers usually contain FFPs and RBCs. Thus, when the demand for blood products genuinely reaches massive amounts, the initial imbalance progressively fades away, and the final ratio converges to 1:1 to 1:2.

**Table 3:** Variability in the composition of transfusion packs among different institutions. Adopted from Hsu et al.^71^

Publication	1^st^ pack	2^nd^ pack	3rd pack
Dente et al. (2009)	6 RBC, 4 FFP	5 RBC, 6 FFP, 1 PC(aph)	6 RBC, 6 FFP, 10 cryo
Riskin et al. (2009)	6 RBC, 4 FFP, 1 PC(aph)	Repeat 1^st^ pack	Repeat 1^st^ pack
Nunez et al. (2010)	10 RBC, 6 FFP, 2 PC(aph)	Repeat 1^st^ pack	Repeat 1^st^ pack
Tan et al. (2012)	4 RBC, 4 FFP	4 RBC, 4 FFP	4 RBC, 4 FFP, 2 PC(aph)
Ball et al. (2013)	6 RBC, 6 FFP	6 RBC, 6 FFP, 1 PC(aph)	6 RBC, 6 FFP, 20 cryo
Bawazeer et al. (2012)	6 RBC, 4 FFP, 1 PC(aph)	4 RBC, 4 FFP	Repeat 2^nd^ pack
Maciel et al. (2015)	6 RBC, 4 FFP, 1 PC(aph)	RBC: FFP (1:1) plus cyo & PC(aph)	Repeat 2^nd^ pack

RBC, Red Blood Cell Concentrates; FFP, Fresh Frozen Plasma units; PC(aph), Platelet Concentrate obtained by apheresis; cryo, Cryoprecipitate.

AB FFP units are considered the “universal type” owing to the absence of anti-A and anti-B antibodies. However, AB is the blood group with the lowest frequency in the population, and AB shortages are a common challenge. An alternative is the use of group-A FFP with low anti-B titers,^88^ since type A individuals are more prevalent, and their plasma is compatible with that of A and O patients (i.e., most of the population). To reduce the risk of hemolysis in type B and AB patients, the type A FFP units used for emergency transfusions have, in most protocols, anti-B titers of up to 100.^89^ In addition, most patients do not ultimately receive massive amounts of incompatible plasma. Furthermore, as FFP is essentially RBC free, RhD compatibility is unnecessary.^90^

Even though platelets express the ABH antigen and preformed anti-A and/or anti-B antibodies can reduce platelet increases (e.g., group-A donors and group-O recipients), ABO compatibility is not a requirement for PC transfusion. However, in individuals with lower blood volumes, minor ABO incompatibility PC transfusions (e.g., group-O donors and group-B recipients) can cause hemolytic reactions from the passive administration of anti-A or anti-B.^90^ For this reason, it seems more prudent to avoid minor incompatibility when transfusing PCs in children.

In MHPs, FFP is the primary source of fibrinogen. However, its low concentration in 1:1:1 or 1:1:2 combinations delays normalization and results in higher levels, such as those required in obstetric hemorrhages.^91^ Thus, the transfusion of cryoprecipitate is essential for patients with hypofibrinogenemia during severe bleeding, as it has higher fibrinogen concentrations than FFP and causes minimal dilution. However, its six-hour expiration time after thawing makes it prone to waste, which is why cryoprecipitates are rarely included in the initial pack. In MHPs with cryoprecipitate empirical use (i.e., without laboratory results), the thawing process starts after activation, and the bags are included in the second or third packs.^83^

While the MHP remains activated, the TS continues to prepare blood units for the next pack, remaining one cycle ahead until the protocol is deactivated.

Laboratory support is also crucial in identifying the need for further transfusions and monitoring coagulation changes. Therefore, rapid and repeated sample collection is essential. Proper labeling, such as that used for TS samples, helps reduce errors and minimize response times. Fast-track protocols are effective methods for expediting results in critical care settings. These protocols typically involve identifying urgent samples, minimizing transport times, and ensuring that laboratory teams are readily available for immediate testing. By implementing these strategies, the results can be expedited, which helps reduce the time spent on “blind transfusions” and ensures more personalized transfusion care, improving patient outcomes.^78^

### MHPs: Misuses and failures

To enable the implementation of MHPs, some requirements ‒ which frontally collide with traditional hemostatic support ‒ are necessary, such as access to blood products without compatibility tests and without laboratory results supporting their actual need. However, these demands increase the possibility of unnecessary transfusions. Therefore, clinicians should use their best judgment to identify situations in which blind transfusions bring more benefits than risks. In this manner, MHPs should not be interpreted as a means through which to obtain prompt access to blood products or an excuse to refrain from sending samples for testing. Additionally, they should remain activated exclusively during the time required to achieve a certain degree of hemodynamic stabilization or until laboratory results allow the transition to a laboratory-guided transfusion strategy.

## MHPs: Case studies

### Case 1

A male patient was involved in a car-bike accident and was transported by ambulance to a tertiary trauma center. Upon arrival, his BP was 70/30 mmHg, his heart rate was 146 bpm, and he presented with a partial amputation of his left leg. An improvised tourniquet had been applied above the injury. During transport, he received 3000 mL of saline, with no medications administered until his arrival. The emergency team activated the MHP, and samples were collected for blood gas analysis, complete blood count and thromboelastometry. The patient was immediately transferred to the operating room, but due to the uncertainty regarding the timing of the trauma, TXA was not administered promptly. The TS provided the first package, which included 4 RBCs and 4 FFP. Initial thromboelastometry results revealed decreased factor levels and hypofibrinogenemia, and 10 units of cryoprecipitate were requested. Approximately 40 minutes later, thromboelastometry indicated fulminant hyperfibrinolysis, and TXA was then administered. During surgery, the bleeding was controlled, the patient's BP stabilized, and MHP was deactivated. The patient was admitted to the ICU and was discharged for rehabilitation three weeks after admission.

### Case 2

A pregnant woman with three previous cesarean sections was admitted to labor. During delivery, the placenta could not be removed, and the patient began to bleed profusely. Samples were collected for complete blood count and conventional clotting tests. Owing to the severity of bleeding and hemodynamic instability, the surgical team decided to perform a hysterectomy. After 3 RBC transfusions and waiting 30 minutes for laboratory results, one unit of platelets and one unit of plasma were blindly requested, which arrived 50 minutes later. The patient was then transferred to the ICU of a reference hospital, where she arrived 2 hours after hemorrhage had started. Her BP was 60/30 mmHg, and her heart rate was 146 bpm. The ICU team activated the MHP, and samples were collected for blood gas analysis, hemoglobin, platelets, PT, aPTT and fibrinogen. The patient received TXA, a first transfusion round with 2 RBCs and a second round with 4 RBCs and 4 FFP, and the initial laboratory results indicated a platelet count of 110,000 mm^3^, a hemoglobin of 6.5 g.dL^−1^, a pH of 7.1 and a lactate level of 12.9 mmoL.L^−1^. Forty minutes later, by the end of the second round, she was more stable, and coagulation tests were completed, revealing a PT of 60 seconds, an aPTT ratio of 2.8 and a fibrinogen level of 43 mg.dL^−1^. MHP was deactivated, and a total of 25 units of cryoprecipitate were transfused. Subsequent samples demonstrated marked improvement, and the patient's bleeding ceased. While her BP stabilized, she did not regain consciousness after sedation was suspended, and her pupils were asymmetrical. A brain CT scan was performed, revealing diffuse swelling and pontine herniation.

## Areas of controversy and future directions

Many areas of controversy persist in the management of major bleeding, starting with the optimal blood product and transfusion protocol. WB may have advantages over separate blood components but remains an area of active research regarding its efficacy. An expiration interval of only 14–21 days and the need to identify donors with low ABO antibody titers may impose logistical difficulties.^78^ Other research areas include the universal use of O RhD-positive WB.^82^

Early treatment of acquired hypofibrinogenemia has been shown to improve outcomes in many bleeding scenarios.^92^, ^93^, ^94^ However, the impact of the source of fibrinogen on patient outcomes remains unclear.^95^ Additionally, questions regarding the composition of fibrinogen concentrates, particularly concerning FXIII contents, need to be investigated.^95^ It also remains uncertain whether fibrinogen should be supplemented early and blindly in MHPs or only when diagnostic tests indicate low levels.^96^

Furthermore, different blood products continue to be investigated, particularly in trauma. Lyophilized plasma, for example, offers logistical advantages^97^ but requires reconstitution and is not available in Brazil.

Another area of study is the optimal dosage of TXA.^40^ Some new trials have evaluated the use of only the first gram of TXA^98^^,^^99^ to overcome the logistical challenges associated with administering an infusion during transport. Preliminary results from a population pharmacokinetic analysis suggest that a 2g bolus followed by a repeated dose 3-hours later is most likely to maintain adequate concentrations in adult patients and should be considered for those with ongoing hemorrhage.^100^ Clinical studies of this dosage are still pending.

Other areas of uncertainty include the ongoing debate between balanced “blinded” transfusion and viscoelastic-guided approaches, identifying which patients benefit from prehospital transfusion, optimizing logistical strategies for its administration and evaluating the effectiveness of various scoring systems in predicting severe blood loss.

## Conclusion

DCR is considered the new paradigm of care in situations with a risk of hemorrhagic death, and MHPs are often adopted as one of its key aspects. This new model fundamentally disrupts traditional bleeding management. With its applications spanning emergency departments, interventional radiology, operating rooms, and military settings, it is essential for all involved professionals to understand the core principles of these protocols and actively engage in their development, implementation, and management.

## Authors’ contribution

DSM conceptualized the article, conducted the literature review, drafted the manuscript and coordinated the reviews by co-authors. DMB, LMBC, LEMC and JSM contributed equally to writing and editing the article. All authors approved the final manuscript as submitted.

## Conflicts of interest

The authors declare no conflicts of interest.

## Appendix Supplementary materials

Image, application 1

## Footnotes

Supplementary material associated with this article can be found in the online version at doi:10.1016/j.bjane.2024.844583.
