# Supplementary material for: A comprehensive review of massive transfusion and major hemorrhage protocols: origins, core principles and practical implementation
Source: Braz J Anesthesiol. 2024 Dec 25;75(2):844583. doi: 10.1016/j.bjane.2024.844583 (PMC11808514; doi:10.1016/j.bjane.2024.844583)
Supplement: Supplementary file 1 [file mmc1.docx]

**BJAN-D-23-00560_ Supplementary Material**

**Supplementary Material** - Simulator

| **Solutes** | | | | **V_Plasma (mL)_** | | | | **V_Non-plasma (mL)_** | | | | **V_Dilution (mL)_** | | | | **Combinations** | | | | | **Expected profile of reconstituted final product** | | | | | | |
| --- | --- | --- | --- | --- | --- | --- | --- | --- | --- | --- | --- | --- | --- | --- | --- | --- | --- | --- | --- | --- | --- | --- | --- | --- | --- | --- | --- |
| **Fib_Cryo_** | **Fib_FFP_** | **Plt_Total_** | **RBC_(mL)_** | **FFP** | **PC** | **RBC** | **Cryo** | **FFP** | **PC** | **RBC** | **Cryo** | **FFP** | **PC** | **RBC** | **Cryo** | **FFP** | **PC** | **RBC** | **Cryo** | **Fluids** | **VT_(mL)_** | **VT_Non-plasma (mL)_** | **VT_Plasma (mL)_** | **%_FactorConc._** | **Ht** | **Plt_(mm3)_** | **Fib_(g/L)_** |
| 16,6 | 2,7 | 5,5E+10 | 180 | 200 | 40 | 30 | 0 | 50 | 10 | 115 | 15 | 250 | 50 | 325 | 15 | 1 | 1 | 1 | 0 | 0 | 625 | 175 | 270 | 0,607 | 0,288 | 88.000 | 1,64 |
| 16,6 | 2,7 | 5,5E+10 | 180 | 200 | 40 | 30 | 0 | 50 | 10 | 115 | 15 | 250 | 50 | 325 | 15 | 1 | 1 | 2 | 0 | 0 | 950 | 290 | 300 | 0,508 | 0,379 | 57.895 | 1,37 |
| 16,6 | 2,7 | 5,5E+10 | 180 | 200 | 40 | 30 | 0 | 50 | 10 | 115 | 15 | 250 | 50 | 325 | 15 | 1 | 1 | 3 | 0 | 0 | 1275 | 405 | 330 | **0,449** | 0,424 | **43.137** | 1,21 |
| 16,6 | 2,7 | 5,5E+10 | 180 | 200 | 40 | 30 | 0 | 50 | 10 | 115 | 15 | 250 | 50 | 325 | 15 | 1 | 2 | 1 | 0 | 0 | 675 | 185 | 310 | 0,626 | 0,267 | *162.963* | 1,69 |
| 16,6 | 2,7 | 5,5E+10 | 180 | 200 | 40 | 30 | 0 | 50 | 10 | 115 | 15 | 250 | 50 | 325 | 15 | 1 | 3 | 1 | 0 | 0 | 725 | 195 | 350 | 0,642 | 0,248 | *227.586* | 1,73 |
| 16,6 | 2,7 | 5,5E+10 | 180 | 200 | 40 | 30 | 0 | 50 | 10 | 115 | 15 | 250 | 50 | 325 | 15 | 2 | 1 | 1 | 0 | 0 | 875 | 225 | 470 | 0,676 | **0,206** | 62.857 | 1,83 |
| 16,6 | 2,7 | 5,5E+10 | 180 | 200 | 40 | 30 | 0 | 50 | 10 | 115 | 15 | 250 | 50 | 325 | 15 | 3 | 1 | 1 | 0 | 0 | 1125 | 275 | 670 | 0,709 | **0,160** | **48.889** | 1,91 |
| 16,6 | 2,7 | 5,5E+10 | 180 | 200 | 40 | 30 | 0 | 50 | 10 | 115 | 15 | 250 | 50 | 325 | 15 | 2 | 2 | 1 | 0 | 0 | 925 | 235 | 510 | 0,685 | **0,195** | *118.919* | 1,85 |
| 16,6 | 2,7 | 5,5E+10 | 180 | 200 | 40 | 30 | 0 | 50 | 10 | 115 | 15 | 250 | 50 | 325 | 15 | 3 | 3 | 1 | 0 | 0 | 1225 | 295 | 750 | 0,718 | **0,147** | *134.694* | 1,94 |
| 16,6 | 2,7 | 5,5E+10 | 180 | 200 | 40 | 30 | 0 | 50 | 10 | 115 | 15 | 250 | 50 | 325 | 15 | 1 | 2 | 2 | 0 | 0 | 1000 | 300 | 340 | 0,531 | 0,360 | *110.000* | 1,43 |
| 16,6 | 2,7 | 5,5E+10 | 180 | 200 | 40 | 30 | 0 | 50 | 10 | 115 | 15 | 250 | 50 | 325 | 15 | 1 | 3 | 3 | 0 | 0 | 1375 | 425 | 410 | **0,491** | 0,393 | *120.000* | 1,33 |
| 16,6 | 2,7 | 5,5E+10 | 180 | 200 | 40 | 30 | 0 | 50 | 10 | 115 | 15 | 250 | 50 | 325 | 15 | 2 | 1 | 2 | 0 | 0 | 1200 | 340 | 500 | 0,595 | 0,300 | **45.833** | 1,61 |
| 16,6 | 2,7 | 5,5E+10 | 180 | 200 | 40 | 30 | 0 | 50 | 10 | 115 | 15 | 250 | 50 | 325 | 15 | 3 | 1 | 3 | 0 | 0 | 1775 | 505 | 730 | 0,591 | 0,304 | **30.986** | 1,60 |
| 16,6 | 2,7 | 5,5E+10 | 180 | 200 | 40 | 30 | 0 | 50 | 10 | 115 | 15 | 250 | 50 | 325 | 15 | 3 | 3 | 1 | 0 | 0 | 1225 | 295 | 750 | 0,718 | **0,147** | *134.694* | 1,94 |
| 16,6 | 2,7 | 5,5E+10 | 180 | 200 | 40 | 30 | 0 | 50 | 10 | 115 | 15 | 250 | 50 | 325 | 15 | 4 | 4 | 1 | 0 | 0 | 1525 | 355 | 990 | 0,736 | **0,118** | *144.262* | 1,99 |
| 16,6 | 2,7 | 5,5E+10 | 180 | 200 | 40 | 30 | 0 | 50 | 10 | 115 | 15 | 250 | 50 | 325 | 15 | 1 | 1 | 1 | 1 | 0 | 640 | 190 | 270 | 0,587 | 0,281 | 85.938 | 2,13 |
| 16,6 | 2,7 | 5,5E+10 | 180 | 200 | 40 | 30 | 0 | 50 | 10 | 115 | 15 | 250 | 50 | 325 | 15 | 1 | 1 | 2 | 1 | 0 | 965 | 305 | 300 | **0,496** | 0,373 | 56.995 | 1,75 |
| 16,6 | 2,7 | 5,5E+10 | 180 | 200 | 40 | 30 | 0 | 50 | 10 | 115 | 15 | 250 | 50 | 325 | 15 | 1 | 1 | 1 | 0 | 250 | 875 | 425 | 270 | **0,388** | **0,206** | 62.857 | 1,05 |
| 16,6 | 2,7 | 5,5E+10 | 180 | 200 | 40 | 30 | 0 | 50 | 10 | 115 | 15 | 250 | 50 | 325 | 15 | 1 | 1 | 1 | 0 | 500 | 1125 | 675 | 270 | **0,286** | **0,160** | **48.889** | **0,77** |
|  |  |  |  |  |  |  | 0 |  |  |  |  | 0 | 0 | 0 |  |  |  |  |  |  | 0 | 0 | 0 | **#DIV/0!** | **#####** | **#DIV/0!** | **#####** |
| ***COMMENTS*** | | | | | | | | | | | | | | | | | | | | | | | | | | | |
| ***1. The expected profile of reconstituted final product was generated from a strictly mathematical analysis. Due to variability in production techniques and the levels of blood elements among donors, the composition and volumes of these;products may oscillate intra- and interinstitutionally. Othe relevant aspecs* in vivo *were also not considered, such as the losses that may occur during storage and jeopardize the recovery tax of each integrant.*** | | | | | | | | | | | | | | | | | | | | | | | | | | | |
| ***2. Adopted charcateristics of blood products are exposed on the table (due to several reasons, this slightly varies between blood banks);*** | | | | | | | | | | | | | | | | | | | | | | | | | | | |
| ***3. Formulas eventually used for calculations may be checked by clicking on the table cells;*** | | | | | | | | | | | | | | | | | | | | | | | | | | | |
| ***4. Pink-filled fields on the last line of table may be edited according to to local characteristics of blood produtcs and the combination to be simulated;*** | | | | | | | | | | | | | | | | | | | | | | | | | | | |
| ***5. Adopted definitions are listed below;*** | | | | | | | | | | | | | | | | | | | | | | | | | | | |
| ***DEFINITIONS*** | | | | | | | | | | | | | | | | | | | | | | | | | | | |
| **Fib_Cryo_** | Concentration, in g.L^-1^, of fibrinogen in Cryoprecipitate (multiply by 100 for the concentration in mg.dL^-1^); | | | | | | | | | | | | | | | | | | | | | | | | | | |
| **FibFFP** | Concentration, in g.L^-1^, of fibrinogen in Fresh Frozen Plasma (multiply by 100 for the concentration in mg.dL^-1^); | | | | | | | | | | | | | | | | | | | | | | | | | | |
| **Plt_Totals_** | Total platelet count in one unit of single (random) Platelet Concentrate (see manuscript for different presentations of platelet concentrates); | | | | | | | | | | | | | | | | | | | | | | | | | | |
| **RBC_(mL)_** | Volume, in mL, occupied by erythrocytes (*i.e.*, erythrocyte volume) in one unit of Red Blood Cells Concentrate; | | | | | | | | | | | | | | | | | | | | | | | | | | |
| **V_Plasma (mL)_** | Volume, in mL, of plasma in each blood component; | | | | | | | | | | | | | | | | | | | | | | | | | | |
| **V_Non-Plasma (mL)_** | Volume, in mL, of non-plasma fluids in each blood component (additive solutions in some RBC's or solutions for anticoagulation and preservation); | | | | | | | | | | | | | | | | | | | | | | | | | | |
| **V_Dilution (mL)_** | Total volume, in mL, of each blood component; for FFP, PC and Cryoprecipitate, it is the sum of plasma and non-plasma volumes; for RBC's, it also includes the erythrocyte volume; | | | | | | | | | | | | | | | | | | | | | | | | | | |
| **FFP** | One unit of Fresh Frozen Plasma; | | | | | | | | | | | | | | | | | | | | | | | | | | |
| **PC** | One unit of single (random) Platelet Concentrate (see manuscript for different presentations of platelet concentrates); | | | | | | | | | | | | | | | | | | | | | | | | | | |
| **RBC** | One unit of Red Blood Cells Concentrate; | | | | | | | | | | | | | | | | | | | | | | | | | | |
| **Cryo** | One unit of Cryoprecipitate; | | | | | | | | | | | | | | | | | | | | | | | | | | |
| **Combinations** | Combination of blood components (in units) and, eventually, asanguineous fluids (in mL); (see manuscript for counting of different presentations of platelet concentrates); | | | | | | | | | | | | | | | | | | | | | | | | | | |
| **Fluids** | Volume, in mL, of asanguineous fluids (essentially crystalloids, colloids and intravenous drugs) used during each "pack" of the evaluated combination; | | | | | | | | | | | | | | | | | | | | | | | | | | |
| **VT_(mL)_** | Total volume, in mL, of the *whole* evaluated combination; | | | | | | | | | | | | | | | | | | | | | | | | | | |
| **VT_Non-plasma (mL)_** | Total volume, in mL, of *non-plasmatic fluids* in the evaluated combination; | | | | | | | | | | | | | | | | | | | | | | | | | | |
| **VT_Plasma (mL)_** | Total volume, in mL, of *plasma* in the evaluated combination; | | | | | | | | | | | | | | | | | | | | | | | | | | |
| **%_FactorsConc._** | Final percent of clotting factors in comparison with the concentration of the original donated plasma (*i.e.*, the plasma contained in the whole blood bag soon after donation); | | | | | | | | | | | | | | | | | | | | | | | | | | |
| **Ht** | Final hematocrit of the evaluated combination; | | | | | | | | | | | | | | | | | | | | | | | | | | |
| **Plt_(mm3)_** | Final platelet count, by mm^3^, of the evaluated combination; | | | | | | | | | | | | | | | | | | | | | | | | | | |
| **Fib_(g/L)_** | Fibrinogen concentration, in g.L^-1^, in the liquid phase (*i.e.*, in the non-erythrocyte volume) of the evaluated combination (multiply by 100 for the concentration in mg.dL^-1^); | | | | | | | | | | | | | | | | | | | | | | | | | | |
